# The Therapeutic Effects of Purified Cortrophin Gel on Experimental Autoimmune Uveitis

**DOI:** 10.1080/09273948.2025.2532821

**Published:** 2025-07-22

**Authors:** Andrew W. Taylor, Madeleine Kim, John L. Zhao, Tat Fong Ng

**Affiliations:** Department of Ophthalmology, Chobanian & Avedisian School of Medicine, Boston University, Boston, Massachusetts, USA

**Keywords:** Adrenocorticotropic hormone, experimental autoimmune uveitis, immunosuppression, melanocortin-based therapy, repository corticotropin gel

## Abstract

**Purpose:**

The melanocortin pathways are central in maintaining the normal anti-inflammatory microenvironment of the eye. A repository corticotropin injection (RCI) from ANI Pharmaceuticals that activates multiple melanocortin pathways was studied for its effects on experimental autoimmune uveitis (EAU), a murine model of human endogenous uveitis.

**Methods:**

At the chronic phase of EAU, the mice received an injection of the RCI. Clinical scoring of the eyes was conducted every 3 to 4 days using fundus microscopy until the uveitis resolved. The eyes were collected, sectioned, and H&E stained for histological scoring. A T-cell reaction assay was performed using spleen cells to measure IFN-g, IL-17, and IL-10 following restimulation with retinal antigen. The effects of the RCI’s active pharmaceutical ingredient (API) on LPS-stimulated macrophage production of IL-1β, IL-10, and TNF-α were assessed by ELISA.

**Result:**

The RCI in a dose-dependent manner suppressed the clinical scores of EAU, showing no significant difference in scoring between 400 U/kg of RCI and a group of native α-MSH-treated mice. In parallel, the retinas of the treated mice had a significant decrease in the histology scores. A significant reduction in IFN-γ and IL-17 production was observed in spleen T-cells of the treated EAU mice, with no regulatory immunity to retinal antigens. The API significantly suppressed the production of IL-1β, IL-10, and TNF-α by stimulated macrophages.

**Conclusions:**

RCI treatment effectively suppressed uveitis and protected the retina from inflammatory damage. These findings further illustrate the potential of melanocortin-based therapies for uveitis.

In a normal eye, the neuropeptide α-MSH suppresses the activation of inflammation and promotes immune regulation.^1–3^ This neuropeptide is consistently present in the eye and has an affinity for four of the five melanocortin receptors (MCRs), and the MCRs are found on cells within the eye.^3–6^ Together with adrenocorticotropic hormone (ACTH), which binds to all five MCRs, beta-MSH, gamma-MSH, and the MCRs, form the melanocortin system that regulates melanogenesis, food intake, energy, sexual function, memory, and behavior. The anti-inflammatory activity mediated by melanocortins is independent of glucocorticoid induction, as demonstrated by α-MSH suppressing inflammation through the MCRs on immune cells.^7–12^ Additionally, α-MSH cannot induce glucocorticoid production since it lacks affinity for MCR2, exclusively expressed in the adrenal cortex with only one ligand, ACTH.^2^ When mice with experimental autoimmune uveitis (EAU) are treated with α-MSH or MCR agonists, the EAU is suppressed, ocular immune privilege is restored, and retinal antigen immune tolerance is induced.^11–14^ This suggests that melanocortin-based therapies can effectively suppress endogenous chronic or steroid-resistant uveitis with potentially long-term protection from recurrence.

Endogenous uveitis is an inflammatory disorder of the uveal tract that is presumed to be autoimmune. It is the third leading cause of visual impairment worldwide, with a prevalence of 17–300 cases per 100,000 individuals.^15–17^ Due to chronic inflammation and tissue damage, uveitis causes visual loss in 28–59% of affected patients. Standard care for managing uveitis includes steroid-based therapies, along with newer non-steroidal options, such as targeted biologics like anti-TNFα antibody infusions.^2,18–20^ While these therapeutic methods effectively reduce inflammation, they can also lead to significant side effects, including immunosuppression, ocular hypertension, and glaucoma.^2,21^ Approximately 50% of uveitis patients treated with steroids experience a recurrence, with 17% developing chronic uveitis that necessitates non-steroidal anti-inflammatory therapy.^15,22,23^ These limitations underscore the urgent need for more effective therapies with improved safety profiles. Melanocortin-based treatments have the potential to fulfill the demand for effective, well-tolerated, steroid-sparing therapy for chronic and steroid-resistant uveitis.^2,3,24,25^

Purified Cortrophin Gel (PCG), a repository corticotropin injection (RCI), is a porcine-derived purified corticotropin made up of a complex mixture of ACTH, ACTH-related peptides and other porcine pituitary-derived peptides approved by the U.S. FDA for treating various inflammatory diseases, including uveitis. While there are several reports on the effectiveness of RCI in suppressing the symptoms of uveitis,^26–29^ little is known about its actions, and it has not been tested in animal models of endogenous uveitis. Therefore, we tested the ability of RCI to suppress EAU and whether it acted like other types of melanocortin receptor agonists by protecting the retina, inducing retinal-antigen-specific Treg cells in the spleen, and activating anti-inflammatory activity in macrophages.^7^ The results showed the RCI suppressed EAU and protected the retina without activating regulatory or anti-inflammatory immune cells.

## Methods

### EAU and Treatment

Experimental autoimmune uveitis (EAU) was induced in 6–8-week-old C57BL/6J mice (Jackson Laboratories, Bar Harbor, ME) as we have done before.^7,8,11,13,30–32^ The mice were housed in the Boston University Animal Science Center, which is AAALAC-certified. The use of the mice was approved by the Boston University Institutional Animal Care and Use Committee and followed the Association for Research in Vision and Ophthalmology statement on the use and care of animals in vision research. The EAU was initiated by subcutaneously injecting the mice with the emulsification of synthesized peptide of human interphotoreceptor retinoid-binding protein (IRBPp) amino acid residues 1–20 of (GenScript, Piscataway, NJ) and complete Freund’s adjuvant (BD Difco, Sparks, MD). In addition, an intraperitoneal injection of pertussis toxin (Sigma, St Louis, MO) was given with a second pertussis toxin injection 2 days later. The retinas were visually assessed using microscopic fundus exams every 3 or 4 days. The severity of retinal inflammation was scored on a 0–5 scale, as previously described.^7,8,14,32–34^ The score was 0 for the eyes with no inflammation; 1 for only white focal lesions of vessels; 2 for linear vessel lesions over less than half of the retina; 3 for linear vessel lesions over more than half of the retina; 4 for severe chorioretinal exudates or retinal hemorrhages in addition to the vasculitis; and 5 for a subretinal hemorrhage or a retinal detachment. There were three independent mouse treatment cohorts with five mice per cohort.

When the mice reached the level of chronic uveitis (sustained level of score 3 around Day 37 after immunization), they were injected subcutaneously with RCI (40 or 400 U/ml, ANI Pharmaceuticals Inc., Baudette, MN) or gel carrier (ANI Pharmaceuticals Inc.). One group of mice was treated with an intraperitoneal injection of native α-MSH (50 μg, Bachem Americas, Inc., Torrance, CA), and with a second injection of α-MSH 2 days later. The concentration of RCI to use was determined by first treating EAU mice with 0, 4, 40, 160, or 400 U/kg (Supplemental Figure 1). The 40 U/kg and 400 U/kg gave a range of effects for the RCI. The retinas were scored until the uveitis began resolving in the α-MSH-treated mice around Day 42 after treatment.

### Histology

The eyes were fixed in 4% paraformaldehyde for 48 h, embedded in paraffin, and cut into 5 μm sections centered on the optic nerve. The sections were stained with hematoxylin and eosin. The stained sections were viewed with a C × 33 microscope (Olympus Shinjuku City, Tokyo, Japan) and QColor 5 camera system (Olympus) and histopathologically scored using an established histopathological scoring criterion.^35^ There were six parameters used to identify the injury conditions of the retina: tissue damage, infiltration, retinal folds, granulomas, vasculitis, and subretinal neovascularization. Each eye was scored from 0 to 4, with 0 for no change, 0.5 for mild, and 1 to 4 relative to the extent of the pathology.

### Spleen cell activation assay

Ng^7^ Mice were immunized for EAU and treated with 0 (gel only), 40, or 400 U/kg RCI. When the 400 U/kg mice resolved, the spleen cells were collected and made into a single-cell suspension, depleted of red blood cells using RBC lysis buffer (Sigma, St Louis, MO), washed and suspended in serum-free media (SFM): RPMI 1640 (Biowhittaker, Walkersville, MD), 10 mM HEPES (Biowhittaker), 1 mM sodium pyruvate (Biowhittaker), nonessential amino acids (Bio Whittaker), 0.2% ITS + 1 (Sigma), 0.1% BSA (Sigma), and 10 μg/mL gentamicin (Sigma) (REF). The spleen cells (4×10^5^ cells/well) were placed in a 96-well round bottom plate, and 50 μg of IRBPp were added to the wells (REF). The cultures were incubated for 48 h at 37°C and 5% CO_2_. The culture supernatant was assayed by ELISA (R&D Systems, Minneapolis, MN) for IFN-γ, IL-17A, and IL-10.

### In vitro macrophage treatment

Kawanaka and Taylor^5^ Peritoneal cells were collected from naive mice by peritoneal lavage. Into each well of a 24-well culture plate, 4 × 10^5^ peritoneal cells in 0.500 ml of RPMI1640 + 10% FBS were added. The cultures were incubated at 37°C for 1–2 h for the macrophages to adhere. The nonadherent cells were removed from the cultures by gently washing the cultures three times with a warm phosphate-buffered solution (PBS). To the cultures were added 0.490 ml of serum-free media (same as above) and 10 μl of 100x active pharmaceutical ingredient (API). The final concentrations of the API were 0, 2.5, 5, 10, 20, 40, and 80 U/ml. The cultures were incubated for 30 min, and then 1 μg/ml LPS in .5 ml of Serum-free media was added to the cultures. The cultures were incubated for an additional 48 h, and the culture supernatants were assayed by ELISA (R&D Systems) for TNF-α, IL-1β, and IL-10.

### Statistics

Statistical analysis was performed using GraphPad Prism 10.4, with p-values ≤ 0.05 considered significant and q ≤ 5% considered a discovered difference. The measurements were performed on 15 mice for EAU scores and histology on 13–15 eyes per group. For the spleen cell assay, 10 spleens per RCI treatment, and 4 independent cultures per API dose for the macrophage assays. The EAU score curves were analyzed using a nonparametric multiple comparison Mann–Whitney test with a two-stage Benjamini, Krieger, and Yekutieli false discovery method post-test. The final EAU scores on Day 42 were analyzed using a one-way nonparametric Kruskal–Wallis test with a Dunn’s multiple comparison post-test. The ELISA data were analyzed with an ordinary one-way ANOVA with Dunnett’s multiple comparison post-test.

## Results

### The effects of RCI on EAU

#### EAU scores

**Figure 1.**
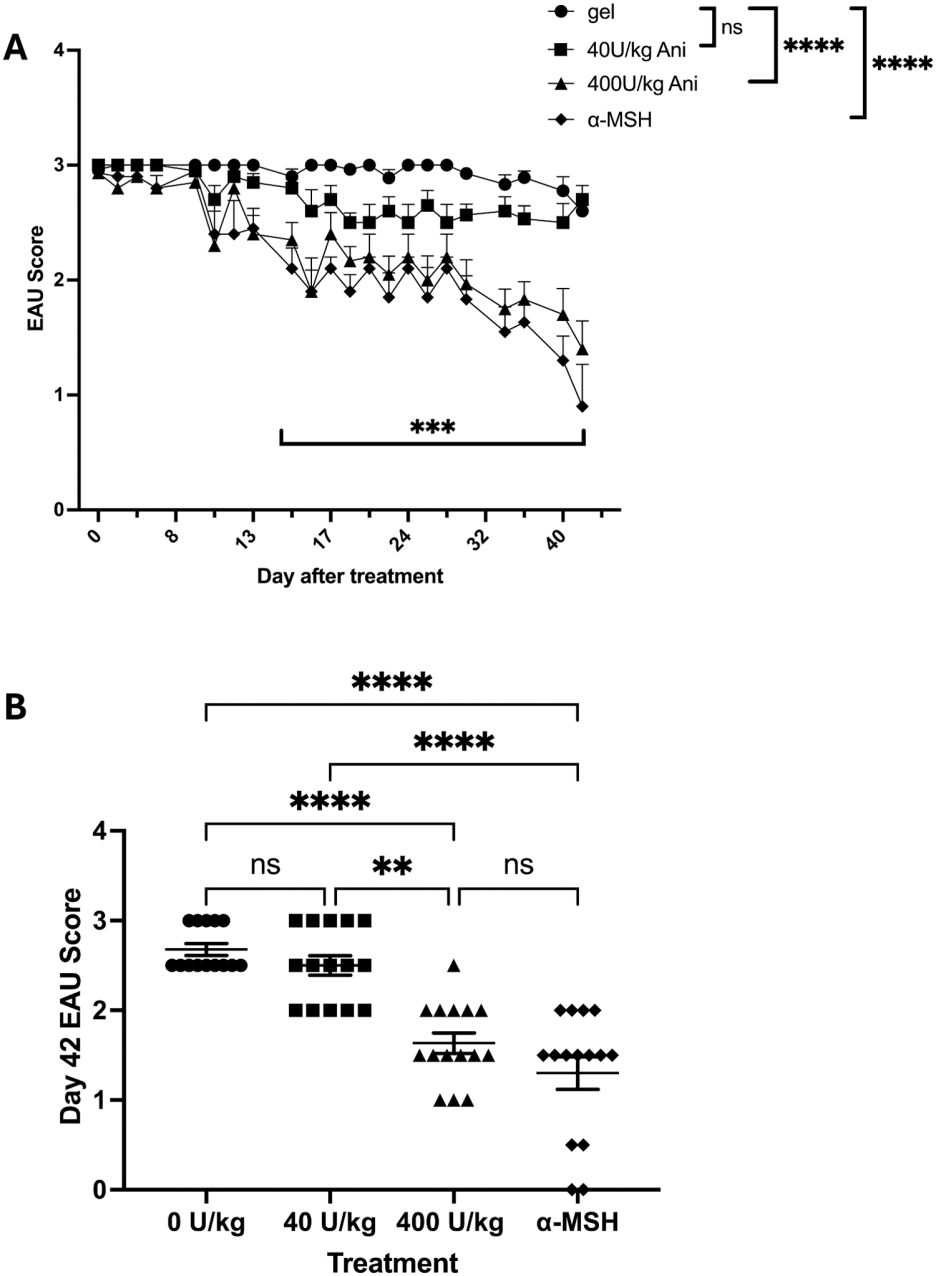
Effects of RCI treatment on EAU. Mice entering the chronic stage of EAU, with a sustained EAU score of 3, were injected with α-MSH (50 μg) or RCI at 0 (Gel Carrier), 40, or 400 U/kg. (A) The mouse eyes were continued to be scored until the α-MSH treated mice reached an average EAU score of 1. Each mouse eye was scored, and the maximum score was used per mouse. There was a 100% incidence of EAU. Presented are the mean ± Standard Error of the Mean (SEM) for each group of mice (*N* = 15 per group). There was a significant suppression in the scores of α-MSH and 400 Ukg RCI from 0 to 40 U/Kg. The EAU scores were significantly lower (***p ≤ 0.005) than the 0 U/kg gel-treated mice on Day 15 for 400 U/kg RCI and α-MSH-treated EAU mice. (B) The final EAU score on Day 42 when the eyes were collected for histology. The mean ± SEM for each mouse in each group shows a significant suppression in EAU scores between 0 U/kg treated mice and 400 U/kg and α-MSH-treated EAU mice. **p ≤ 0.01, ****p ≤ 0.001, ns = no statistical difference.

Previous studies have shown that activating the melanocortin system with melanocortin receptor ligands such as α-MSH suppresses the severity and duration of EAU.^7^ Since the RCI primarily comprises ACTH-based peptides that are melanocortin receptor agonists, we evaluated its effects on EAU. The mice were immunized to induce EAU, and their eyes were clinically assessed through fundus examination. When the mice reached the chronic phase of EAU, marked by a sustained clinical score of 3, they were injected with 400, 40, or 0 (vehicle control) U/kg of RCI. For a positive control group, mice were injected with α-MSH (50 μg).^7^ EAU scoring continued until uveitis began to clear in the α-MSH-treated mouse group 42 days following RCI treatment, reaching an average EAU score of 1 (Figure 1A). The clinical scores over time in mice treated with 400 U/kg or α-MSH were significantly (*p* ≤ 0.001) different from the EAU scores of the mice treated with 40 or 0 U/kg. No statistical difference was found in the EAU scores between the mice receiving 400 U/kg and those treated with α-MSH.

However, sustained statistical differences in scores between 0 U/kg gel-injected and α-MSH-treated 400 U/kg treated EAU mice were reached at 15 Days. There was no statistical difference in the EAU scores between the groups receiving 40 U/kg and 0 U/kg. These statistical differences are seen in the individual EAU scores of the mice in each group at the end of the experimental run Day 42 (Figure 1B). The results demonstrated RCI suppression of EAU.

#### Histology

**Figure 2.**
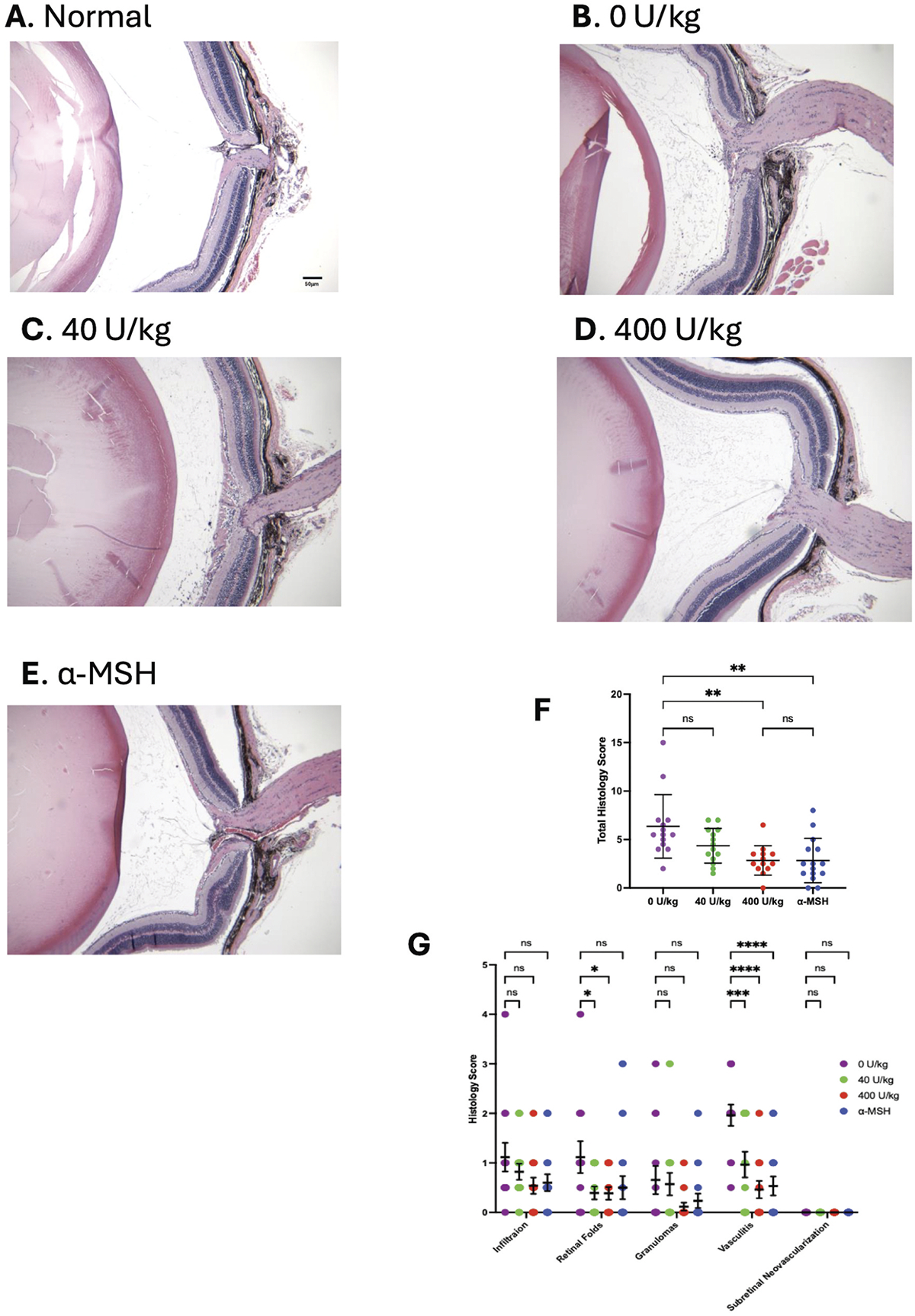
Effects of RCI treatment on retinal structure. The eyes collected on day 40 were sectioned and H&E stained. Representative images of the stained sections from (A) Normal, (B) untreated EAU mice (0 U/kg), or mice treated with (C) 40 U/kg RCI, (D) 400 U/kg RCI, (E) native α-MSH peptide. The sections were scored. (F) The total histology core is provided for each mouse per group (*N* = 13 per group). Normal mouse eyes have scores equal to zero. Statistical differences (** *p* ≤ 0.01) exist between the EAU mice treated with 400 U/kg RCI or α-MSH and the untreated 0 U/kg gel. There were no statistical differences (ns) between 40 U/kg RCI and untreated 0 U/kg gel. (G) Scores of the six individual criteria per group showed significant discovery (*d ≤ 0.05, **d ≤ 0.01, ****d ≤ 0.001) of differences between the RCI-treated groups in retinal folds and vasculitis.

The eyes were collected 42 days after treatment from the mice, sectioned, stained with H&E, and retinal pathology scored. Overall, visual examinations of the eyes show that 400 U/kg RCI treatment, compared to normal eyes, preserved the retina, possibly better than an α-MSH treatment (Figure 2(A,D,E)). The total histopathology score of the retinas of mice treated with 400 U/kg significantly differed (*p* ≤ 0.01) from the histopathology scores of 40 and 0 U/kg RCI (Figure 2F). There was no statistical difference between 400 U/kg RCI and α-MSH-treated EAU mice. The individual histopathology criteria scores show that RCI treatment suppressed the incidences of retinal folds and vasculitis even better than α-MSH treatment (Figure 2E). These results demonstrated the effectiveness of RCI, at least at the 400 U/kg dose, suppressing EAU and preserving the retina during uveitis.

### RCI-treatment of EAU mice on IRBP-specific T cell activity

**Figure 3.**
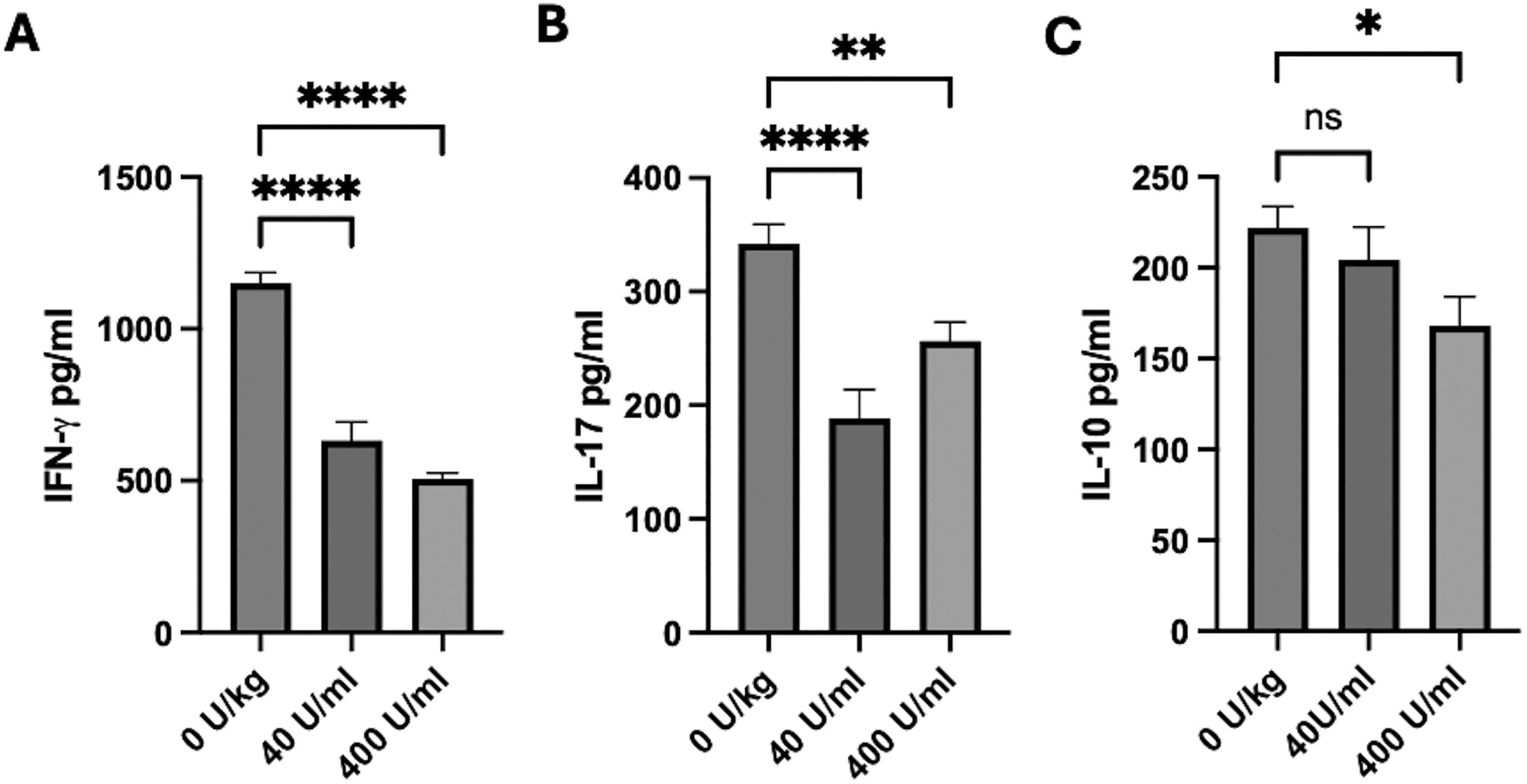
Effects of RCI treatment on retinal antigen-specific spleen T cells. Spleen immune cells were collected 42 days after treatment and cultured with IRBPp, and 48 h later, the culture media was assayed by ELISA (A) IFN-γ, (B) IL-17, and (C) IL-10. There was significant (**p ≤ 0.01, ****p ≤ 0.001, *N* = 10) suppression in IFN-γ and IL-17 in the EAU mice treated with the RCI. While there was no significant (ns) difference in IL-10 between untreated (0 U/kg) and 40 U/kg RCI-treated EAU mice, there was significant (*p ≤ 0.05) suppression in IL-10 production by the spleen T cells of mice treated with 400 U/kg.

Previous publications have shown that activating the melanocortin system to accelerate the resolution of EAU results in retinal-antigen-specific T cells that produce IL-10 (Treg cells) in contrast to uveitic T cells producing IFN-γ and IL-17 in the spleen.^11^ To see if RCI treatment induces Treg cell responses to IRBP in the spleen, the immune cells from the EAU mice were collected 42 days after the EAU mice were treated with 400, 40, or 0 U/kg of RCI. After 48 h of in vitro antigen stimulation, the culture supernatant was assayed for IFN-γ, IL-17, and IL-10 (Figure 3). There was a significant decrease in IFN-γ and IL-17 levels in the T cell cultures. The RCI suppressed the T cell response to retinal antigen but did not activate Treg cell activity.

### The effects of the active pharmaceutical ingredient (API) on cytokine production by activated macrophages

**Figure 4.**
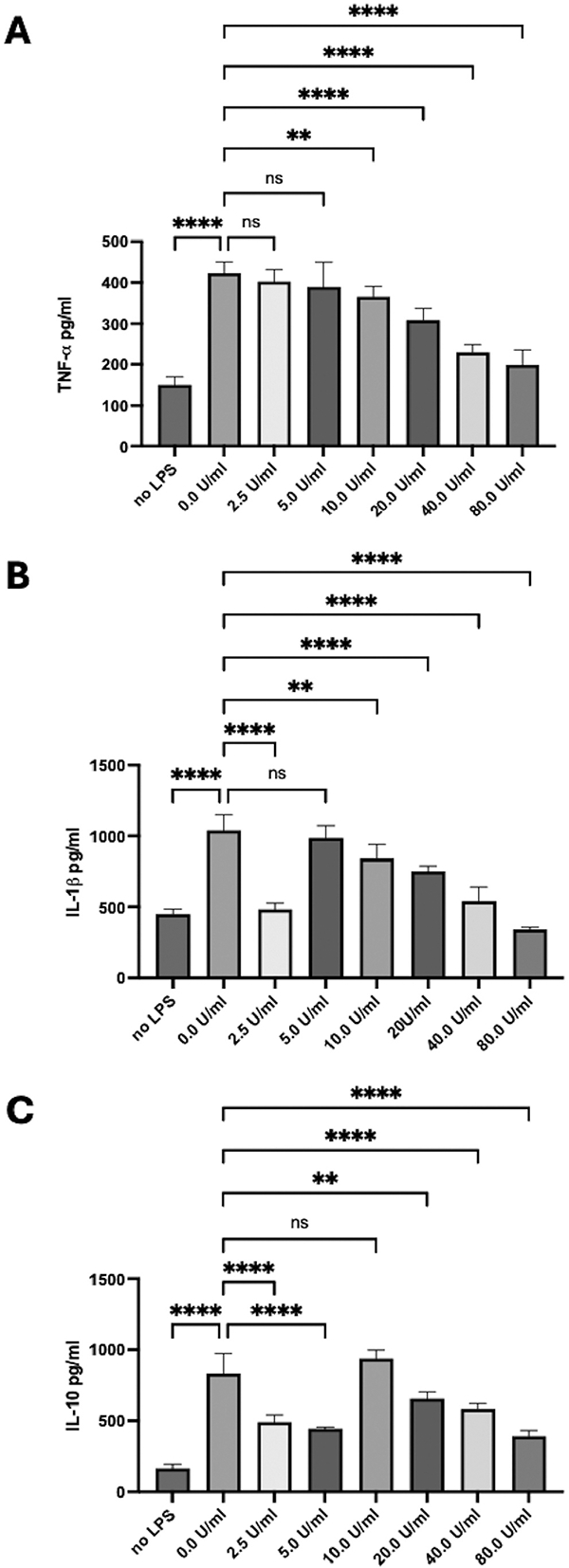
Effects of API on LPS-stimulated macrophages. Cultured macrophages were treated with API (0, 2.5, 5, 10, 20, 40, and 80 U/ml) and then with LPS (1 μg/ml). After 48 h, the culture media was assayed by ELISA for TNF-α, IL-1β, and IL-10. The API significantly (**p ≤ 0.01, ****p ≤ 0.001, *N* = 4) suppressed the production of TNF-α in a dose-dependent manner and a monophasic response for IL-1β and IL-10 with higher concentrations of API suppressing cytokine production. ns = not statistically different.

Peritoneal macrophages were activated in culture and treated with the API at 0–80 U/ml. After 48 h of incubation, the supernatants were assayed for TNF-α, IL-1β, and IL-10 (Figure 4). Significant increases in all three cytokines were observed in the activated macrophage cultures; however, TNF-α production was significantly suppressed in a dose-dependent manner (Figure 4A). A monophasic suppressive response was seen with IL-1β production at concentrations greater than 5 U/ml (Figure 4B). There was an apparent monophasic suppression of IL-10 production with the minimum effect centered on 10 U/ml (Figure 4C). These results demonstrated that at the higher concentrations of the API, corresponding with the initial injection of RCI, there was suppression of macrophage activity with no induction of suppressor cells or anti-inflammatory activity by the macrophages.

## Discussion

The results demonstrate that treatment with the RCI effectively suppresses EAU and protects the retina against inflammation-induced damage. In contrast to reports that used α-MSH or α-MSH-peptide analogs that target melanocortin receptors but not the melanocortin 2 receptor (MCR2),^36,37^ the RCI and its active pharmaceutical ingredients suppress immune cell activity without inducing regulatory immunity. The benefit must be through stimulation of melanocortin receptors in the retina,^4,7,38^ in contrast to the additional actions of MSH to induce anti-inflammatory activity in macrophages and Treg cells.^5,12,32,39^ This suggests the RCI gel works more like ACTH in suppressing immune cell activity more through suppressing immune cell activity, along with the benefits of protecting the retina through melanocortin-stimulated pathways.

The central action of the Hypothalamic-Pituitary-Adrenal (HPA) Axis is ACTH, which, through the MCR2 on adrenal glands, induces glucocorticoid release.^37,40^ The systemic elevation of glucocorticoids in the bloodstream can suppress inflammation and is the basis of synthetic corticosteroid treatment for inflammation. While ACTH stimulation of glucocorticoid production may be part of an ACTH-administered therapy, treatment with ACTH protein binds all five melanocortin receptors, potentially providing additional benefits and, in some cases, countering the adverse effects of corticosteroid treatment.^41^ Purified ACTH has been effectively used as a treatment for multiple sclerosis.^42,43^ Along with suppressing inflammation, which is presumed to be through glucocorticoid production, there is stimulation of MC4r on the preganglionic sympathetic nervous system, mediating the release of noradrenalin that suppresses immune cell production of cytokines.^42^ Oral forms of ACTH treatment in mouse models of MS increase the frequency of Treg cells in the gut, thereby suppressing inflammation.^9^ Also, ACTH treatment protects bones from osteonecrosis induced by glucocorticoid therapy.^41^ These suggest that using an ACTH-based treatment, like the RCI gel, has both the anti-inflammatory effects of glucocorticoid elevation and the benefits of activating the melanocortin system. Our results demonstrated that the RCI effectively suppressed immune activity with protection of the retina in EAU mice.

Therapeutic application of α-MSH into EAU mice results in suppression of uveitis, preservation of retinal structure, and induction of splenic suppressor APCs and autoantigen-specific Treg cells.^30,32,44–46^ This response is absent of glucocorticoid stimulation since the native neuropeptide α-MSH binds to the other four MCRs but not MCR2. A differential immunoregulatory reaction is suggested depending on the pattern of MCRs stimulated. Suppression of innate immune-mediated inflammation by α-MSH appears mostly through MCR1 with MCR3.^7,39,47^ With MCR5 stimulation, α-MSH mediates the induction of suppressor APCs that counter-convert autoantigen-specific effector T cells into inducible T cells.^8,48,49^ Preserving retinal structure and cell survival needs MCR1, MCR4, and MCR5 stimulation during inflammation and diabetic retinopathy.^7,50–55^ Our results demonstrate that treatment with the RCI gel suppressed EAU through general suppression of immune cell activity without Treg cell development while protecting the retina. Therefore, the effects of RCI therapy must occur through the stimulation of melanocortin receptors, in conjunction with the actions of glucocorticoids, making RCI function more similar to ACTH in suppressing inflammation than α-MSH, which works by altering the activity of immune cells. This also proposes the potential of selecting melanocortin peptides that choose different melanocortin receptors to regulate innate immune activity, induction of Tregs, or general suppression of immune cell activity and inflammation. Another effect of the selective melanocortin receptor-agonist activity is the preservation of retinal structure and retinal cell survival.

The use of RCI for treating severe acute and chronic allergic and inflammatory processes involving the eye and its adnexa is approved by the U.S. FDA. The potential of RCI gels to treat non-infectious uveitis has been reported in several clinical trials.^26,28,29,56,57^ These studies and literature reviews indicate that RCI-gel is a highly effective anti-inflammatory and autoimmune therapy. The side effects of an ACTH-induced corticosteroid spike, such as hypertension, were reported as minimal or tolerable. However, a retrospective study involving a small cohort of six patients with ocular sarcoidosis indicated that they were withdrawn from treatment due to lack of benefit and experienced side effects from the RCI-gel therapy.^58^ The reasons for this group’s intolerable side effects remain unclear, particularly since it has been shown that the levels of hydroxycorticosteroids in the blood of patients treated with intravenous steroids are 20-fold greater than in those treated with the RCI-gel.^42^ The suppression of inflammation and the results of our EAU treatment with RCI-gel may be related to ACTH’s ability to stimulate the full range of melanocortin receptors, resulting in both the resolution of uveitis and the preservation of the retina.

The suppression of EAU with α-MSH or MCR-agonists results in the establishment of ocular immune privilege and immune tolerance to ocular autoantigens, providing a potentially long-term resistance to the recurrence of uveitis.^7,12^ While the application of the RCI-gel suppresses EAU, whether this is associated with the reestablishment of ocular immune privilege and an immunosuppressive microenvironment will have to be studied. The lack of expansion of Treg cells in the spleen suggests that the RCI-gel treatment does not mediate the induction of systemic immune tolerance to ocular autoantigens. How this may or may not provide long-term protection from the recurrence of uveitis is to be seen. Our results showed that there is an effective suppression of EAU by RCI-gel treatment with the potential to provide an alternative therapy to steroid-resistant and chronic uveitis.

## Supplementary Material

Figure S1

Supplemental data for this article can be accessed online at https://doi.org/10.1080/09273948.2025.2532821
