# Supplementary figures and images for: The Therapeutic Effects of Purified Cortrophin Gel on Experimental Autoimmune Uveitis
Source: Ocul Immunol Inflamm. Author manuscript; Available in PMC 2025 Jul 31. (PMC12313274; doi:10.1080/09273948.2025.2532821)

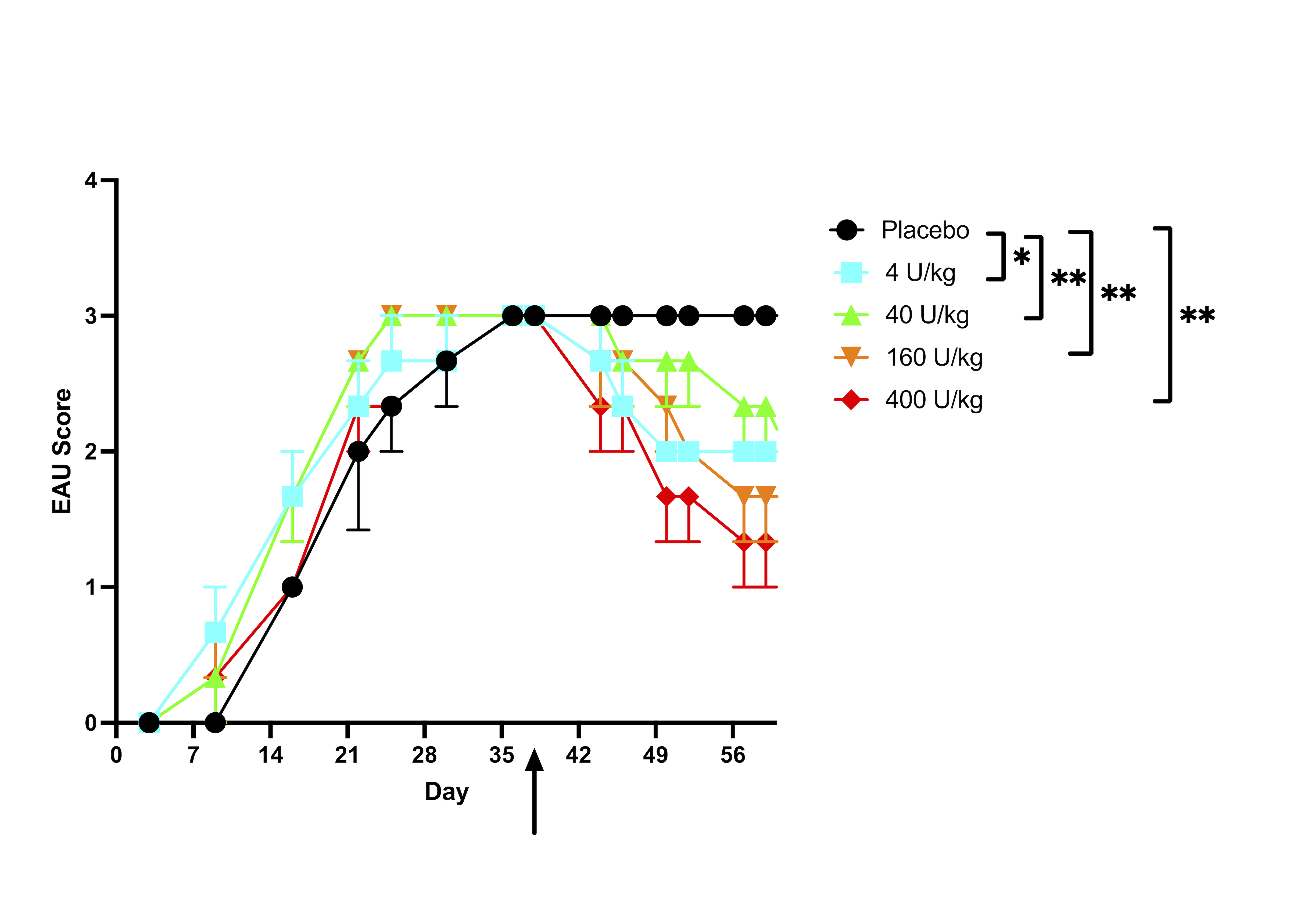

Supplement: Figure S1 [file NIHMS2099405-supplement-Figure_S1.tiff]
